# Clinical characteristics and macrolide resistance analysis of pertussis in children: a retrospective study

**DOI:** 10.3389/fpubh.2026.1821827

**Published:** 2026-07-10

**Authors:** Huifen Zhang, Xiaoqing Yang, Jiaowei Guo, Jiming Li

**Affiliations:** 1 Department of Pediatrics, Women and Children's Hospital, School of Medicine, Xiamen University, Xiamen, China; 2 Department of Medical Laboratory, Fujian Key Clinical Specialty of Laboratory Medicine, Women and Children's Hospital, School of Medicine, Xiamen University, Xiamen, China

**Keywords:** pertussis, infants, leukocytosis, macrolide resistance, vaccine

## Abstract

**Purpose:**

Pertussis is mainly caused by *Bordetella pertussis*. Currently, no reports exist on the incidence of *B. pertussis* infection or on its patterns of macrolide resistance among pediatric cases in Xiamen, Fujian Province, China. The aim of this study was to determine the clinical characteristics and macrolide resistance patterns in pediatric pertussis.

**Methods:**

We retrospectively collected data from 968 children with pertussis between February 2021 and January 2025. Demographic characteristics, disease trends, clinical features, and macrolide resistance among outpatients and inpatients were analyzed.

**Results:**

Most inpatients with pertussis were infants aged < 3 months, whereas most outpatients were school-aged children. Inpatients demonstrated significantly higher rates of leukocytosis, fever, co-infection, recent exposure to individuals with a cough, comorbid pneumonia, and macrolide use and experienced longer time from cough onset to confirmed diagnosis than did outpatients. The proportion of children who presented with spasmodic cough, violent coughing with facial flushing, inspiratory whoop, tachypnea, cyanosis, hypoxemia, and respiratory failure, as well as the average hospital stay, was significantly higher among children with severe pertussis than among those with common pertussis. Age younger than 3 months, being unvaccinated, co-infection, leukocytosis, lymphocytosis, spasmodic cough, facial flushing, inspiratory whoop, tachypnea, and cyanosis were risk factors for severe pertussis. Macrolides were the main antibiotics prescribed for children with pertussis. The detection rate of drug-resistant mutation sites was 77.52% in outpatients and 92.50% in inpatients.

**Conclusion:**

It is necessary to enhance the vaccination of pertussis for special populations, including women in the later pregnancy stages and school-aged children. Children with pertussis who have risk factors should be monitored to identify severe cases. The empirical use of macrolides for pertussis treatment is effective in most cases; alternative agents should be considered if the response is suboptimal, especially when drug-resistant mutation sites are identified.

## 1 Introduction

Pertussis is a respiratory infectious disease mainly caused by *Bordetella pertussis* (1). Typical pertussis is characterized by a paroxysmal spasmodic cough, an inspiratory whoop, and peripheral blood lymphocytosis. Severe pertussis may lead to life-threatening complications, including pneumonia, encephalopathy, and pulmonary hypertension (2). Since the 1980s, some developed countries with high vaccine coverage rates have reported a resurgence of pertussis, and this public health problem has also emerged in China (3). Children represent the highest risk group for pertussis. Prompt diagnosis and intervention can reduce transmission, prevent progression to severe disease, and mitigate the burden of pertussis. In 1994, the first macrolide-resistant strain of *B. pertussis* was identified in the United States (4). The complete genome sequence of a macrolide-resistant *B. pertussis* strain from Japan was published in 2010 (5). These findings confirmed that the isolate harbored homologous A2047G mutations in all three copies of its *23S rRNA* gene, a genotype commonly observed in macrolide-resistant strains of *B. pertussis* from China (5, 6). Recent reports have indicated a high prevalence of macrolide resistance among *B. pertussis* isolates from diverse regions of China (6). Genome-based analysis showed that the genotype MT28-*ptxP3*-MRBP rapidly dominated after 2020; the MT28-*ptxP3*-MRBP cluster was identified in France, Japan, and the United States in 2024, indicating potential cross-border transmission (7). To date, macrolide antibiotics remain the first-line treatment for pertussis. Currently, no reports exist on the incidence of *B. pertussis* infection or on its macrolide resistance patterns among pediatric cases in Xiamen, Fujian Province, China. This study aimed to determine the clinical characteristics and macrolide resistance of pertussis in children, thereby providing a valuable basis for clinicians to improve the diagnosis and treatment of pertussis.

## 2 Materials and methods

### 2.1 Study cohort

We retrospectively collected clinical data from pediatric patients with pertussis who received outpatient or inpatient care at the Women and Children’s Hospital, School of Medicine, Xiamen University, between February 2021 and January 2025. The inclusion criteria were as follows: (1) children aged < 14 years, irrespective of sex, and (2) a positive test for *B. pertussis* DNA by real-time quantitative fluorescence polymerase chain reaction (Kit: *Bordetella pertussis* Nucleic Acid Detection Kit; Manufacturer: Shenzhen Yilifang Biotech Co., Ltd.). No exclusion criteria were applied. This study was approved by the Medical Ethics Committee of our hospital (No. KY-2025-107-K01) and was conducted in accordance with the principles of the Declaration of Helsinki. The requirement for informed consent was waived owing to the retrospective nature of the study.

### 2.2 Diagnostic criteria for severe pertussis

Severe pertussis was defined as meeting at least one of the following criteria: recurrent apnea, hypoxemia, respiratory failure, pertussis encephalopathy, white blood cell count ≥ 30 × 10^9^/L with deteriorating clinical symptoms, concurrent pulmonary arterial hypertension, sepsis, single or multiple organ dysfunction (8).

### 2.3 Case data collection

The following data were collected: (1) demographic characteristics, including sex, age at disease onset, and seasonality of onset; (2) clinical parameters, including exposure to individuals with a cough, pertussis vaccination history, time from cough onset to confirmed diagnosis, antibiotic administration after diagnosis, clinical manifestations, therapeutic interventions, and prognostic outcomes; (3) laboratory investigations, including the results obtained from *B. pertussis* nucleic acid testing, detection results of drug-resistant mutation sites of *B. pertussis* (A2047G mutation in the *23S rRNA* gene of *B. pertussis*), full blood count, and serum levels of C-reactive protein (CRP) and lactate dehydrogenase (LDH); and (4) imaging studies, including chest radiography or pulmonary computed tomography and echocardiography.

### 2.4 Statistical analyses

SPSS software (version 22.0; IBM, Armonk, NY, United States) was used for data analysis. Non-normally distributed quantitative data are expressed as medians (Q1, Q3), and the Mann–Whitney *U* test was used for intergroup comparisons. Categorical data are expressed as percentages (%). The chi-square test or Fisher’s exact probability method was used to compare categorical variables. Statistical significance was set at *p* < 0.05. Binary logistic regression analysis was used to identify risk factors associated with severe pertussis in children.

## 3 Results

### 3.1 Demographic characteristics

Overall, 968 participants were included: 859 outpatients and 109 inpatients. The age distribution, sex composition, and seasonal patterns among children with pertussis are shown in Table 1.

**Table 1:** Demographic characteristics of pertussis in children.

Characteristic	Outpatients with pertussis (*n* = 859), %	Inpatients with pertussis (*n* = 109), %
Age		
<3-month infants	11 (1.28)	61 (55.96)
≥3-month infants	77 (8.96)	31 (28.44)
Toddlers	64 (7.45)	2 (1.83)
Preschool children	152 (17.69)	6 (5.50)
School-age children	555 (64.61)	9 (8.26)
Sex		
Male	485 (56.46)	57 (52.29)
Female	374 (43.54)	52 (47.71)
Season		
Spring	361 (42.03)	43 (39.45)
Summer	249 (28.99)	26 (23.85)
Autumn	61 (7.10)	19 (17.43)
Winter	188 (21.89)	21 (19.27)

### 3.2 Number of pediatric pertussis cases per month

**Figure 1:**
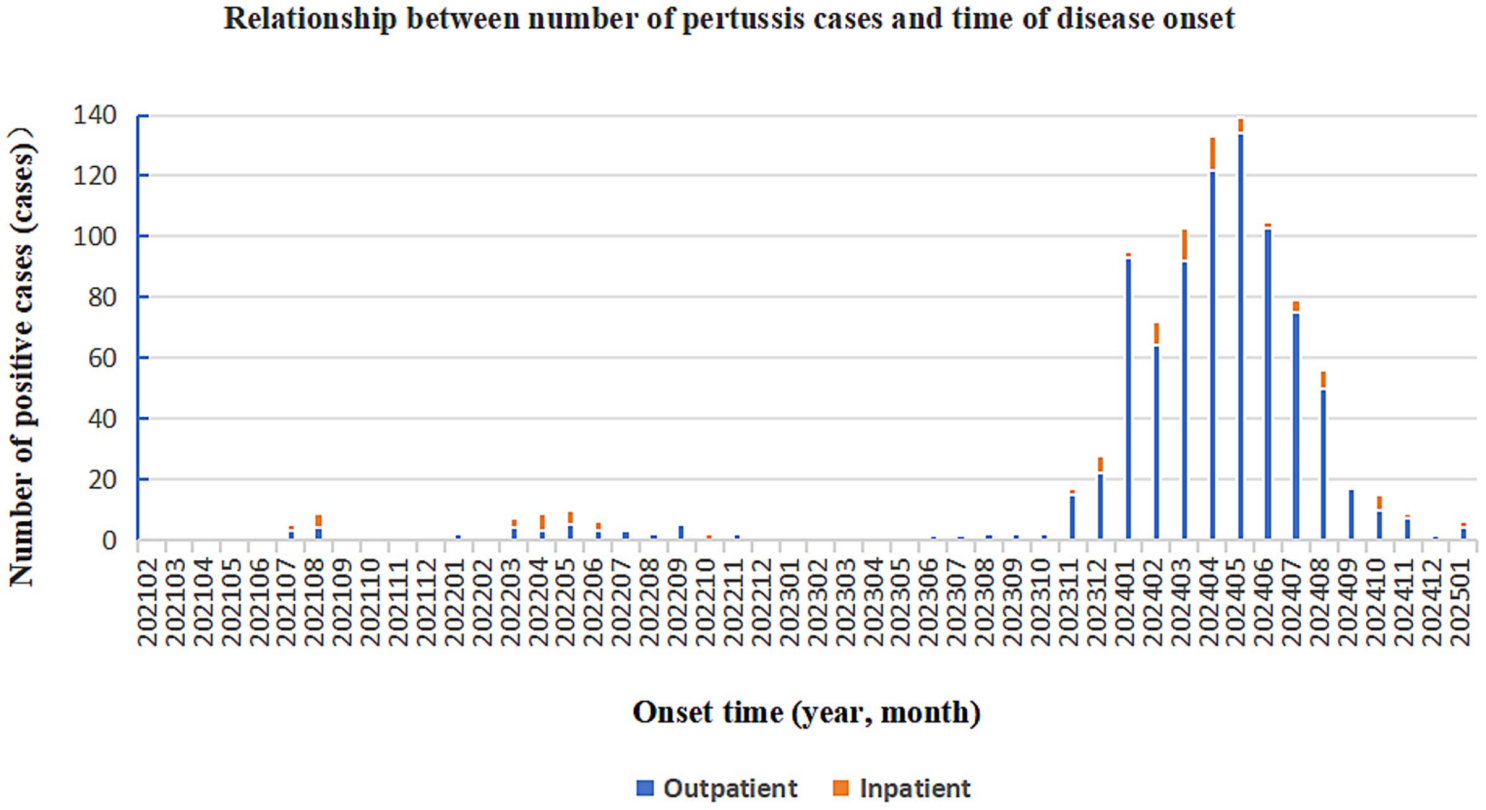
Relationship between number of pertussis cases and time of disease onset (February 2021–January 2025).

A distinct outbreak phase was observed between November 2023 and October 2024, with sporadic cases occurring throughout the remaining period of the 4-year cycle spanning February 2021 to January 2025 (Figure 1).

### 3.3 Comparison of clinical characteristics between outpatients and inpatients

The proportions of hospitalized children with leukocytosis, fever, co-infection, exposure to individuals with a cough, concomitant pneumonia, and use of macrolide antibiotics were higher than those among outpatients, and the differences, except for the difference in elevated CRP (*p* = 0.538), were statistically significant (*p* < 0.05). The time from cough onset to confirmed diagnosis was longer for hospitalized children than for outpatients (Table 2).

**Table 2:** Comparison of clinical characteristics between pediatric outpatients and inpatients.

Variables	Outpatients with pertussis (*n* = 859), %	Inpatients with pertussis (*n* = 109), %	*χ*^2^ or *Z*	*p*-value
Leukocytosis	118/319 (36.99)	75/109 (68.81)	33.216	<0.001
Elevated CRP	32/311 (10.29)	9/109 (8.26)	0.379	0.538
Time from cough onset to confirmed diagnosis (days)^a^	9 (6, 14)	10 (7, 15)	−2.278	0.023
Fever	108/859 (12.57)	23/109 (21.10)	6.012	0.014
Co-infection	35/859 (4.07)	64/109 (58.72)	314.542	<0.001
Exposure to individuals with a cough	21/859 (2.44)	56/109 (51.38)	316.303	<0.001
Concomitant pneumonia	52/224 (23.21)	69/100 (69.0)	61.940	<0.001
Treatment with macrolide antibiotics	473/817 (57.89)	88/109 (80.73)	21.007	<0.001

^a^*M*_median_ (Q1, Q3).

CRP, C-reactive protein.

### 3.4 Comparison of clinical data between common and severe inpatient pertussis

Among 109 hospitalized cases of pertussis in children, 81 were common cases and 28 were severe cases. No statistically significant differences were observed in sex, exposure to individuals with a cough, or time from cough onset to confirmed diagnosis between patients with common and severe pertussis cases (*p* > 0.05). The proportion of children under 3 months of age, unvaccinated, or co-infected among patients with severe pertussis was higher than that among patients with common pertussis (*p* < 0.05). The proportions of children with leukocytosis and lymphocytosis were also significantly higher among patients with severe pertussis than among those with common pertussis (*p* < 0.05); however, no significant differences were observed in CRP or LDH levels (*p* > 0.05). No significant differences were observed in the rates of fever, post-tussive vomiting, concomitant pneumonia, or lung rales between severe and common pertussis cases (*p* > 0.05). The incidence of spasmodic cough, facial flushing, inspiratory whoop, tachypnea, cyanosis, hypoxemia, and respiratory failure, as well as the average length of hospital stay, were significantly higher among severe cases than among common cases, and these differences were statistically significant (*p* < 0.05; Table 3).

**Table 3:** Comparison of clinical data between common and severe inpatient pertussis.

Variables	Common pertussis (*n* = 81), %	Severe pertussis (*n* = 28), %	*χ*^2^ or *Z*	*P*-value
Younger than 3 months	40/81 (49.38)	21/28 (75.00)	5.541^b^	0.027
Male	42/81 (51.85)	15/28 (53.57)	0.025^b^	0.875
Unvaccinated	47/81 (58.02)	23/28 (82.14)	5.267^b^	0.022
Exposure to individuals with a cough	41/81 (50.62)	17/28 (60.71)	0.852^b^	0.356
Co-infection	43/81 (53.09)	21/28 (75.00)	4.122^b^	0.048
Leukocytosis	47/81 (58.02)	27/28 (96.43)	13.948^c^	<0.001
Lymphocytosis	43/81 (53.09)	24/28 (85.71)	9.267^c^	0.003
Elevated CRP	6/81 (7.41)	4/28 (14.29)	1.171^c^	0.276
Elevated LDH	57/69 (82.61)	25/26 (96.15)	2.902^c^	0.105
Time from cough onset to confirmed diagnosis (days)^a^	11 (7, 15)	10 (7, 15)	−0.697^d^	0.486
Fever	16/81 (19.75)	7/28 (25.00)	0.344^b^	0.557
Concomitant pneumonia	50/77 (64.94)	23/28 (82.14)	2.870^b^	0.09
Treatment with macrolide antibiotics	66/81 (81.48)	23/28 (82.14)	0.006^b^	0.938
Average length of hospital stay^a^	6 (4, 9)	9 (7, 11)	−2.780^d^	0.005
Spasmodic cough	54/81 (66.67)	25/28 (89.29)	5.288^c^	0.026
Facial flushing	51/81 (62.96)	25/28 (89.29)	6.767^c^	0.009
Post-tussive vomiting	33/81 (40.74)	14/28 (50.00)	0.727^b^	0.394
Inspiratory whoop	4/81 (4.94)	6/28 (21.43)	6.728^c^	0.017
Tachypnea	1/81 (1.23)	18/28 (64.29)	56.945^c^	<0.001
Cyanosis	13/81 (16.05)	20/28 (71.43)	30.230^b^	<0.001
Lung rales	60/81(74.07)	22/28(78.57)	0.226^b^	0.635
Hypoxemia	1/81 (1.23)	13/28 (46.43)	37.616^c^	<0.001
Respiratory failure	0/81 (0)	11/28 (39.29)	35.069^c^	<0.001

^a^*M*_median_ (Q1, Q3); ^b^chi-square test; ^c^Fisher’s exact probability method; ^d^Mann–Whitney *U* test.

CRP, C-reactive protein; LDH, lactate dehydrogenase.

### 3.5 Risk factors for severe pertussis in hospitalized children

**Figure 2:**
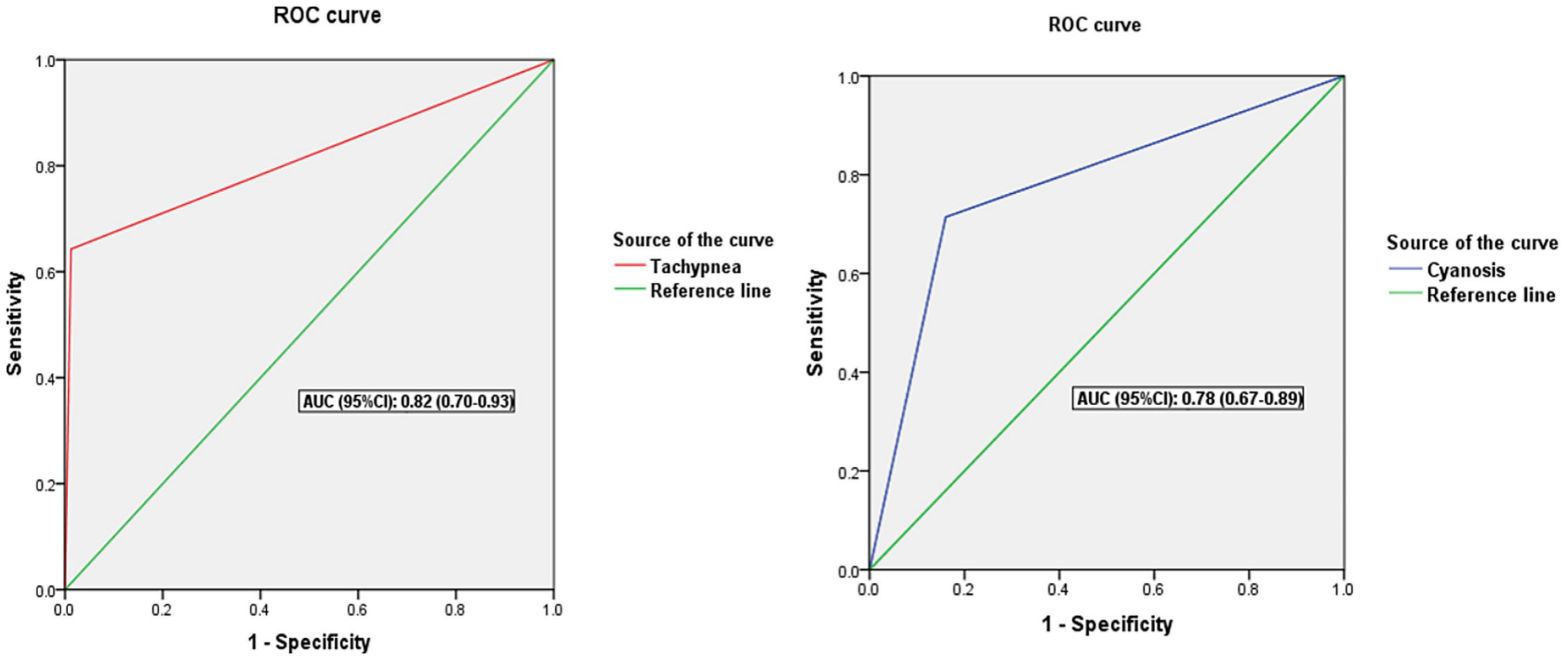
Receiver operating characteristic curve of strong risk factors for severe pertussis in hospitalized children.

By analyzing the clinical data of patients with common and severe pertussis who were hospitalized, we initially identified the potential risk factors for severe pertussis in hospitalized children. Using univariate binary logistic regression analysis, the risk factors for severe pertussis in hospitalized children were identified (Table 4). Diagnostic performance of the risk factors for predicting severe pertussis were shown in Table 5. Tachypnea (AUC = 0.82) and cyanosis (AUC = 0.78) were the two strongest independent risk factors for predicting severe pertussis, Receiver Operating Characteristic (ROC) curves for these risk factors were drawn (Figure 2).

**Table 4:** Univariate binary logistic regression analysis of potential risk factors for severe pertussis in hospitalized children.

Variables	*β*	*P*	OR	95% CI
Younger than 3 months	1.123	0.022	3.075	1.177–8.030
Unvaccinated	1.202	0.027	3.328	1.149–9.634
Co-infection	0.975	0.047	2.651	1.015–6.926
Leukocytosis	2.972	0.004	19.532	2.529–150.833
Lymphocytosis	1.668	0.004	5.302	1.687–16.661
Concomitant pneumonia	0.910	0.097	2.484	0.848–7.274
Spasmodic cough	1.427	0.029	4.167	1.154–15.040
Facial flushing	1.590	0.015	4.902	1.363–17.624
Inspiratory whoop	1.658	0.016	5.250	1.360–20.271
Tachypnea	4.970	<0.001	144.000	17.315–1197.602
Cyanosis	2.571	<0.001	13.077	4.753–35.978

**Table 5:** Diagnostic performance of the risk factors for predicting severe pertussis.

Variables	AUC value	95%CI	*P*-value	Sensitivity (%)	Specificity (%)	Youden Index
Younger than 3 months	0.63	0.51–0.75	0.044	75.00	50.60	0.256
Unvaccinated	0.62	0.51–0.74	0.058	82.10	42.00	0.241
Co-infection	0.61	0.49–0.73	0.085	75.00	46.90	0.219
Leukocytosis	0.69	0.59–0.79	0.003	96.40	42.00	0.384
Lymphocytosis	0.66	0.55–0.77	0.010	85.70	46.90	0.326
Spasmodic cough	0.61	0.50–0.73	0.075	89.30	66.70	0.226
Facial flushing	0.63	0.52–0.74	0.038	89.30	37.00	0.263
Inspiratory whoop	0.58	0.45–0.71	0.195	21.40	95.10	0.165
Tachypnea	0.82	0.70–0.93	<0.001	64.30	98.80	0.631
Cyanosis	0.78	0.67–0.89	<0.001	71.40	84.00	0.554

### 3.6 Macrolide resistance and antibiotic use

Among 859 outpatients with pertussis, 218 were screened for drug-resistant mutation sites, of whom 169 (77.52%) exhibited the A2047G mutation in the *23S rRNA*. Among 109 inpatients, 40 were tested, and a mutation was detected in 37 (92.50%) cases (Table 6).

**Table 6:** Detection of drug-resistant mutation sites and antibiotic use in pediatric pertussis.

Antibiotic use	Outpatients		Inpatients	
	No mutation, %	Mutation, %	No mutation, %	Mutation, %
Administration of macrolide antibiotics	35 (71.43)	116 (68.64)	3 (100)	22 (59.46)
Administration of sulfamethoxazole	14 (28.57)	51 (30.18)	0 (0)	5 (13.51)
Administration of alternative antibiotics due to suboptimal macrolide response	0 (0)	2 (1.18)	0 (0)	10 (27.03)
Total	49	169	3	37

## 4 Discussion

*Bordetella pertussis* is mainly transmitted through the respiratory tract and aerosols, and infections can occur throughout the year. After implementing the pertussis immunization strategy, the incidence rate decreased significantly (9). However, globally, pertussis has re-emerged owing to increased awareness of pertussis among medical staff, improved sensitivity of symptom monitoring, the widespread use of polymerase chain reaction detection, variations in strains of *B. pertussis*, and the inability of natural infection and vaccination to induce lifelong immunity (10).

This study found that inpatients with pertussis were more likely to be aged < 3 months. The proportion of children aged < 3 months among inpatient children with severe pertussis was significantly higher than that among those with common pertussis, a difference that is consistent with the findings of Wang et al. (11). Among outpatients with pertussis, the cases were generally mild, with school-age children comprising the largest proportion of patients, followed by preschool children. This may be because vaccine-induced protection wanes over time, making children susceptible to infection; however, the immune system of older children matures gradually, providing better protection and clearance capacity. If these children are not diagnosed and isolated promptly, they may become sources of infection, warranting clinical attention. The incidence of pertussis was similar between male and female children, with the lowest rates observed in autumn. During the study period, pertussis showed an outbreak trend from November 2023 to October 2024, partially overlapping with the outbreak period (April–July) reported by Cai et al. (12). In the present study, the outbreak period was longer, while cases during the other periods were sporadic, reflecting the high infectivity of the disease.

Fever is a rare clinical feature of pertussis (8); however, in the present study, 23 of 109 hospitalized children presented with fever, and 20 of these cases had concomitant co-infection with other pathogens. The specific pathogens identified were as follows: *human rhinovirus* (*n* = 7), *human parainfluenza virus* (*n* = 4), *human respiratory syncytial virus* (*n* = 4), *Haemophilus influenzae* (*n* = 3), *Mycoplasma pneumoniae* (*n* = 2), *human cytomegalovirus* (*n* = 2), *human bocavirus* (*n* = 1), *Streptococcus pneumoniae* (*n* = 1), and *SARS-CoV-2* (*n* = 1). These co-infecting pathogens were similar to those reported by Gan and Wu (13). In recent years, mixed lower respiratory tract infections have become common among hospitalized children (14). In our study, 84.40% of hospitalized children with pertussis were infants, and the proportion with co-infections (58.72%) was significantly higher than that among outpatients (4.07%). Therefore, clinicians should actively screen for and identify pathogens during clinical management process. Cases of exposure to individuals with a cough were significantly higher among hospitalized children than among outpatients. This finding may be because hospitalized children were mainly infants with limited mobility, and their family members could provide more detailed histories. In contrast, most outpatients were preschool or school-aged children who engaged in a wider range of activities, making source tracing more difficult. The Pertussis Diagnosis and Treatment Plan (2023), issued by the National Health Commission of China, considers a cough lasting ≥ 2 weeks accompanied by paroxysmal spasmodic cough as one of the diagnostic criteria for clinical cases (15). However, in this study, the median time from cough onset to diagnosis was 11 days for outpatients and 12 days for inpatients. Cases with a cough lasting < 2 weeks accounted for approximately 36% of confirmed pertussis cases (16). Therefore, children with a cough lasting < 2 weeks should also be screened promptly in clinical practice.

Vaccine coverage is a key factor in controlling the spread of pertussis (17). Although maternal–fetal antibodies are present in newborns, their half-life is approximately 6 weeks; therefore, the protective maternal antibodies in infants younger than 3 months gradually wane before vaccination (18). In our study, the proportion of unvaccinated children in the severe group was 82.14%, which was significantly higher than that in the common group. Early initiation of vaccination can provide protection for infants, may reduce disease severity after pertussis onset, and can lessen the burden on healthcare resources (19). The administration of the pertussis vaccine during the third trimester of pregnancy has positive implications. Previous research (20) has shown that peripheral blood lymphocyte counts in children with pertussis were generally elevated, particularly in severe cases. Leukocyte levels are strongly correlated with the severity of pertussis, the risk of complications, and mortality (21). The present study showed that the proportions of children with leukocytosis and lymphocytosis were significantly higher among patients with severe pertussis than among those with common pertussis. These differences are clinically valuable for identifying severe disease. In this study, only 36.99% of pediatric outpatients with pertussis presented with leukocytosis; therefore, leukocytosis can only be used as an auxiliary diagnostic indicator. The final diagnosis should be based on clinical symptoms and pertussis pathogen detection. CRP and LDH levels are indicators of systemic inflammation (22, 23). In this study, both common and severe cases showed partial elevations; however, the differences between the groups were not statistically significant, indicating that these indicators cannot be used to predict clinical severity. The proportions of children with spasmodic cough, violent cough with facial flushing, inspiratory whoop, tachypnea, cyanosis, hypoxemia, and respiratory failure, as well as the average length of hospital stay and the proportion requiring ventilatory support, were significantly higher among severe cases than among common cases. These differences were statistically significant and consistent with the findings of Zhang et al. (24). The study showed that infants younger than 3 months, being unvaccinated, co-infection, leukocytosis, lymphocytosis, spasmodic cough, facial flushing, inspiratory whoop, tachypnea, and cyanosis were risk factors for severe pertussis. Tachypnea and cyanosis were the two strongest independent risk factors for predicting severe pertussis. Age is an independent protective factor; younger patients are more likely to require respiratory support (25). Children aged < 3 months who have not received the pertussis vaccine are prone to developing severe pertussis after infection. Treatment is complex, and clinical follow-up for young infants should be strengthened to ensure optimal outcomes.

Macrolide antibiotics are the first-line treatment for pertussis. In this study, 80.73% of hospitalized children with pertussis were initially treated with macrolide antibiotics, and 10 subsequently received other antibiotics. Among outpatients, 57.89% were treated with macrolide antibiotics whereas 39.23% received sulfamethoxazole. To date, the only confirmed mechanism conferring macrolide resistance in *B. pertussis* is a point mutation (A2047G) at position 2047 of the V domain of the *23S rRNA* gene (26). In this study, 258 cases of pertussis were screened for drug-resistant mutation sites. Among the 218 outpatients screened, 169 (77.52%) exhibited A2047G mutation in the *23S rRNA*. Of the 118 who received macrolide therapy, two (1.69%) showed inadequate response and required a change of antibiotic. Thirty-seven (92.50%) inpatient cases exhibited A2047G mutation in the *23S rRNA*, and of the 32 treated with macrolides, 10 (31.25%) required alternative antibiotics. Our findings showed that the mutation detection rate of drug-resistant mutation sites in *B. pertussis* strains was high, and the actual clinical failure rate with macrolide antibiotics was relatively low. The discrepancy between the mutation detection rate and clinical efficacy may be related to the following factors: (1) the anti-inflammatory and immunomodulatory effects of macrolide drugs can alleviate airway inflammation and relieve symptoms (27, 28) and (2) inhalation treatments, such as using budesonide and terbutaline therapy, can also relieve airway spasms, improve cyanosis, and improve breathing difficulties. To prevent the transmission of macrolide-resistant *B. pertussis* strains, we advocate strengthening surveillance for macrolide-resistant *B. pertussis* isolates and implementing alternative therapeutic regimens upon confirmed drug resistance to interrupt the transmission chain. A limitation of this study is its single-center design; therefore, our results may not fully represent the entire picture. Future multi-center studies are needed to better present the clinical characteristics of pertussis in children in the study location.

In conclusion, it is necessary to enhance the vaccination of pertussis for special populations, including women in the later pregnancy stages and school-aged children. Children with pertussis who have risk factors should be monitored to identify severe cases. The empirical use of macrolides for pertussis treatment is effective in most cases; alternative agents should be considered if the response is suboptimal, especially when drug-resistant mutation sites are identified.

## Data Availability

The original contributions presented in the study are included in the article/supplementary material, further inquiries can be directed to the corresponding author.
